# Dynamic Changes in Bilirubin Predict 90-Day Mortality in Patients With Hepatocellular Carcinoma and Acute Decompensations of Cirrhosis: The HCC-AD Score

**DOI:** 10.1016/j.mayocpiqo.2025.100661

**Published:** 2025-09-12

**Authors:** Oliver Moore, Fran Neveu-Coble, Scott Read, Wai-See Ma, Adnan Nagriel, Anna Di Bartolomeo, Jacob George, Golo Ahlenstiel

**Affiliations:** a Blacktown Clinical School, School of Medicine, Western Sydney University, Blacktown, New South Wales, Australia; b Blacktown Hospital, Western Sydney Local Health District, Blacktown, New South Wales, Australia; c Storr Liver Centre, The Westmead Institute for Medical Research, University of Sydney, Westmead, New South Wales, Australia; d Westmead Hospital, Western Sydney Local Health District, Westmead, New South Wales, Australia

## Abstract

**Objective:**

To develop a score to predict 90-day mortality in patients with hepatocellular carcinoma (HCC) admitted with an acute decompensation (AD) event of chronic liver disease.

**Patients and Methods:**

This retrospective cohort study was conducted at Blacktown and Westmead Hospitals in Australia, including patients with decompensated cirrhosis and concomitant HCC between January 1, 2012, and May 31, 2023. Participants were separated into derivation (n=233) and validation (n=132) cohorts. Demographic and clinical data were collected at admission and day 7. Independent predictors for 90-day transplant-free survival were entered into classification and regression tree analysis to develop the HCC-AD score. Discrimination was assessed in the validation cohort using Harrell C statistic. Subgroup analysis was conducted for each Barcelona Clinic Liver Cancer (BCLC) class with comparisons made to current scores.

**Results:**

A cohort of 355 patients was considered. Admission bilirubin (*P*=.009) and 7-day change in bilirubin (*P*=.018) remained significant for 90-day mortality in multivariable analysis. The HCC-acute decompensation (AD) score stratified patients into 3 risk groups with predicted mortality of 26%, 49%, and 89%, respectively. The HCC-AD score showed good discrimination (Harrell C=0.731). Cox regression analysis determined the HCC-AD score remained predictive in BCLC B (*P*<.001), C (*P*<.001), and D (*P*=.010) scored HCC. The model for end-stage liver disease 3.0 (*P*=.058) and Child-Pugh (*P*=.11) scores were not predictive in BCLC D HCC.

**Conclusion:**

A simple score that stratifies patients with HCC into 3 risk categories based on changes in bilirubin predicts 90-day mortality following an acute decompensatory event. It is superior to other scores in advanced HCC.

Mortality rates for patients with hepatocellular carcinoma (HCC) remain high, driven not only by liver tumor burden and metastases but also by underlying liver dysfunction and portal hypertension secondary to cirrhosis.^1^^,^^2^ Prognostication can be difficult due to the competing risks imposed by 2 different underlying pathophysiological processes.^1^^,^^3^ Decompensatory events in advanced chronic liver disease, including ascites, hepatic encephalopathy, and/or variceal bleeding, are predictive of mortality in patients with HCC, irrespective of the tumor burden.^4^ Therein, prediction of mortality in patients with HCC experiencing an acute decompensation of their liver disease may be more robust for stratifying short-term mortality and decision making on ongoing therapeutic interventions.

Current prediction models for assessing survival in HCC routinely used in clinical practice include the Child-Pugh, albumin-bilirubin (ALBI) ratio, and the model for end-stage liver disease (MELD) scores.^5^, ^6^, ^7^ Other verified scores for mortality prediction, such as the Okuda and Cancer of the Liver Italian Program score engage histopathological and radiological variables that may not be readily available in practice.^8^ While there is evidence for the utility of these scores,^5^^,^^9^, ^10^, ^11^ they are static scores that do not consider the recovery of liver function during a patient’s admission for decompensation.^12^ They also do not provide any information on the potential futility of an intervention. The Lille score was developed for early identification of patients with alcoholic hepatitis who may benefit from alternative therapies. Using dynamic changes in bilirubin and other static variables, the authors showed a combination is superior to dynamic and static models individually.^13^^,^^14^ Models of this nature have not been developed for patients with HCC.

Bilirubin has long been appreciated as a measure of underlying hepatic dysfunction, with increases in serum bilirubin known to be an independent predictor of 90-day mortality in patients with cirrhosis.^1^^,^^15^ We hypothesized that a model combining dynamic changes in bilirubin with other static variables is predictive of 90-day transplant-free survival (TFS) in patients with HCC admitted with an acute hepatic decompensation event, irrespective of tumor burden. We hypothesized that this model will be noninferior to current predictive scores such as the MELD 3.0, Child-Pugh, and ALBI scores. Subgroup analysis will be conducted on patients in each Barcelona Clinic Liver Cancer (BCLC) stage. As a secondary outcome, we compared dynamic changes in serum bilirubin with changes in MELD 3.0, Child-Pugh, and ALBI scores to predict TFS.

## Patients and Methods

### Study Design

In this retrospective cohort study, we analyzed the electronic health records of 847 admissions of patients with HCC from Western Sydney Local Health District, Australia, admitted between January 1, 2012 and May 31, 2023. The study was approved by the ethics committee of Western Sydney Local Health District (HREA 2021/ETH00149) and is in accordance with the tenets of the Declaration of Helsinki.

### Participants

The International Classification of Disease 10 codes were used to identify 847 admissions of patients with HCC at Blacktown and Westmead Hospitals. Patients were included if they presented with an acute decompensation of advanced chronic liver disease, in the setting of synchronous HCC. Patients lacking a clear decompensatory event during their admission, with no clinicopathological data available or those who died within 7 days were excluded (Supplemental Figure 1, available online at http://www.mcpiqojournal.org). The development of ascites requiring therapeutic intervention, bleeding esophageal varices, or hepatic encephalopathy were considered decompensatory events. We excluded hospitalizations with previous liver transplant, patients with noncirrhotic HCC, and pediatric patients (below 18 years of age), as well as patients discharged against medical advice or those transferred from another acute care hospital. A derivation cohort of 233 patients was collected from Westmead Hospital, Australia, while a validation cohort of 132 patients were from Blacktown Hospital, Australia. Demographical data, etiology of advanced liver disease, reason for each decompensatory event, and International Classification of Disease 10 codes were recorded for all presentations. Information on each patient’s HCC was collected including BCLC stage, radiological factors, and previous treatment. Laboratory parameters for each patient were collected at admission and day 7, with the interval change derived. MELD 3.0, Child-Pugh, CLIF-C AD, and ALBI scores were calculated using standard medical calculators (https://www.mdcalc.com). The MELD 3.0 score was computed according to the original formula proposed by the Mayo Clinic.

Date of death was obtained from both local health databases and the Australian registry of deaths where available. Mortalities were recorded until August 2023. Liver transplantation was considered an end point. All patients were deidentified.

### Statistical Analyses

Means and SDs were calculated for continuous variables, whereas numbers and percentages were used for categorical variables. For univariable statistical comparisons, the χ^2^ test was used for categorical variables. Student *t* test and Mann-Whitney U test were used for continuous variables.

Univariable logistic regression analysis was conducted on the derivation cohort using laboratory parameters and demographical characteristics. Odds ratios with 95% CIs and regression coefficients were obtained. Listwise deletions were used for missing variables. Variables that achieved significance (*P*<.05) for predicting 90-day mortality were considered for further analysis. Combinations of factors were trialed in classification and regression tree (CART) analysis to develop a simple and meaningful model. Factors included in models were also entered into multivariate analysis to ensure that they remained independently significant for prediction of 90-day mortality. A final model, named the HCC-AD model, which stratified patients into distinct risk groups (groups A-C) was derived from the CART analysis.

Discriminatory performance of HCC-AD was compared with the MELD 3.0, Child-Pugh, and ALBI scores through calculating the area under receiver operating characteristic curve for each score. Harrell C statistic was calculated for each score, with Somersd used to calculate *P* values and 95% CIs comparing 2 nested cox models. Kaplan-Meier analysis was used to evaluate overall survival at 90 days based on the grouping of HCC-AD. Log rank pairwise comparisons were conducted for the HCC-AD score groups to investigate for significantly different survival distributions. Statistical significance was set adjusted to *P*<.0167 using a Bonferroni correction. Calibration of HCC-AD was done by visual confirmation of goodness of fit comparing observed and predicted survival. A multivariate regression including the HCC-AD score and the CLIF-C AD score, was completed to ensure that the predictive capacity of the HCC-AD score remained when adjusting for an independent acuity score.

To examine the effect of tumor stage on HCC-AD’s ability to predict outcome, a logistic regression model was developed. BCLC stage and HCC-AD were used as predictors with *P* value of <.05 considered significant. Subgroup analysis was conducted on the entire cohort, wherein regression models were used to assess whether the HCC-AD score remained prognostic for 90-day mortality in each BCLC stage. The MELD 3.0 and Child-Pugh score were also assessed using regression analysis in each of the BCLC stages. Previous treatment modalities, radiological characteristics of HCC, and the impact of transplant allocation scores (Milan criteria and metroticket survival calculator) were examined using the same method to assess whether tumor-specific variables improved the prognostic capacity of the HCC-AD score. Subgroup analysis accounting for gender and etiology of liver disease to verify the generalizability of the score. Patients experiencing a decompensatory event secondary to progression of HCC were analyzed using regression analysis to determine if the HCC-AD score remained predictive.

Data analyses were performed using Stata 17.0 (Stata Corp LLC) and R Statistical Software (v4.1.2; R Core Team). The study was reported in line with STROBE guidelines.

## Results

Data were collected from 365 patients with HCC presenting with acute decompensation of cirrhosis between January 2012 and May 2023 as summarized in Table 1. The median age of the derivation cohort was 65 (IQR, 60-72) years, and 184 patients (82.50%) were men (Table 1). Chronic hepatitis C virus (HCV) was the most common etiology of HCC, accounting for 100 patients (42.92%), followed by alcohol in 58 patients (24.89%), hepatitis B virus in 44 patients (18.88%), and metabolic dysfunction–associated liver disease in 41 patients (17.60%). The most common decompensatory event was ascites, which was present in 130 patients (55.79%). The median bilirubin at admission in the derivation cohort was 50.00 (IQR, 25-85) μmol/L, whereas the median change in bilirubin after 7 days was 0.0 (IQR, −8 to −13) μmol/L. The median MELD 3.0 and ALBI scores were 19.68 (IQR, 14.37-24.64) and −1.14 (IQR, −1.54 to −0.69). Further, 118 patients (50.64%) died within 90 days of admission. Sodium, creatinine, bilirubin, albumin, international normalized ratio (INR); day 7 sodium, bilirubin, INR, and the 7-day change in bilirubin were significantly different in those with 90-day TFS (Supplemental Table 1, available online at http://www.mcpiqojournal.org). In the validation cohort, 61 patients (46.21%) died within 90 days of admission. Seven patients (5.30%) were lost to follow up within the first 90 days.

**Table 1:** Baseline Characteristics in the Derivation and Validation Cohorts

Characteristics	Derivation set (n=233)	Validation set (n=132)	*P*
Age (y), mean (IQR)	65.00 (12.0)	63.00 (12.0)	<.001
Male sex	184 (82.5)	119 (90.2)	.006
Etiology of liver disease			
EtOH	58 (24.89)	47 (35.61)	.03
HCV	100 (42.92)	55 (41.67)	.82
HBV	44 (18.88)	11 (8.33)	.007
MASH	41 (17.60)	14 (10.60)	.07
Sign of AD at inclusion			
Ascites	130 (55.79)	68 (51.52)	.52
Hepatic encephalopathy	99 (42.49)	31 (23.48)	<.001
Variceal bleeding	30 (12.88)	29 (21.97)	.02
Admission laboratory parameters			
Sodium (mmol/L)	135.00 (7.0)	136.0 (6.0)	.079
Creatinine (mmol/L)	87.00 (56.0)	92.0 (58.0)	.55
Bilirubin (μmol/L)	50.00 (60.0)	31.0 (36.50)	.002
Albumin (mmol/L)	26.00 (7.5)	26.0 (7.50)	.66
INR	1.40 (0.50)	1.30 (0.30)	.045
7d laboratory parameters			
Sodium (mmol/L)	135.00 (6.0)	136.0 (7.0)	.26
Creatinine (mmol/L)	81.00 (54.0)	83.0 (70.0)	.58
Bilirubin (μmol/L)	48.00 (75.0)	28.0 (55.50)	.006
Albumin (mmol/L)	28.00 (7.0)	26.0 (9.0)	.052
INR	1.50 (0.50)	1.40 (0.30)	.005
Change over first 7d			
Sodium (mmol/L)	0 (6.0)	1.0 (7.0)	.11
Creatinine (mmol/L)	−4.00 (29.0)	−3.0 (32.0)	.66
Bilirubin (μmol/L)	0.0 (21.0)	-0.5 (16.50)	.96
Albumin (mmol/L)	1.0 (7.0)	0.0 (5.0)	.32
INR	0 (0.30)	0.00 (0.20)	.35
ALBI score, mean (±SD)			
Admission	−1.14 (0.85)	−1.19 (0.77)	.43
7d	−1.30 (0.92)	−1.24 (0.94)	.54
7d change	−0.09 (0.58)	−0.02 (0.48)	.45
MELD 3.0 score, mean (±SD)			
Admission	19.68 (10.27)	16.20 (9.57)	.002
7d	19.95 (9.57)	16.17 (8.28)	.009
7d change	0.64 (4.16)	0.60 (5.97)	.92
Child-Pugh score, mean (±SD)			
Admission	9.0 (2.0)	8.0 (2.0)	<.001
7d	9.0 (3.0)	8.0 (2.0)	.002
7d change	0.0 (1.0)	0.0 (1.0)	.4
Death during admission	45 (19.31)	32 (24.24)	.35
Time to death (d), mean (±SD)	45.0 (131)	79.0 (246.0)	.01
Mortality			
30d mortality	69 (29.6)	36 (27.27)	.64
90d mortality	118 (50.64)	61 (46.21)	.74
180d mortality	143 (0.61)	77 (58.33)	.57
BCLC class			<.001
A/0	59 (27.06)	44 (33.33)	
B	48 (22.02)	43 (32.57)	
C	100 (45.87)	15 (11.36)	
D	11 (5.05)	30 (22.73)	
Radiological characteristic			
Tumor number	72 (34.78)	42 (43.75)	.062
1	37 (17.87)	24 (25.00)	.014
2	21 (10.14)	5 (5.21)	.023
3	77 (37.20)	25 (26.04)	.59
4 or more	3.0 (3.8)	5.0 (6.10)	.003
Largest tumor size	215 (99.54)	101 (96.19)	
Portal hypertension	95 (45.67)	45 (42.45)	
Tumor-related PVT	47 (22.07)	9 (8.49)	
Extrahepatic metastases			
Previous therapy			
Resection	8 (3.43)	9 (6.82)	.14
Microwave ablation	43 (18.45)	18 (13.64)	.24
TACE	70 (30.04)	36 (27.27)	.58
SIRT	16 (6.87)	7 (5.30)	.56
SBRT	6 (2.58)	2 (1.54)	.51
Chemotherapy	5 (2.15)	0 (0)	.09
Immunotherapy	66 (28.33)	14 (10.61)	<.001

Values are n (%) unless specified.

AD, acute decompensation; ALBI, albumin-bilirubin ratio; BCLC, Barcelona Clinic Liver Cancer; HBV, hepatitis B virus; HCC, hepatocellular carcinoma; HCV, hepatitis C virus; INR, international normalized ratio; MASH, metabolic dysfunction–associated steatohepatitis; MELD, model for end-stage liver disease; PVT, portal vein thrombosis; SBRT, Stereotactic body radiation therapy; SIRT, selective internal radiation therapy; TACE, transarterial chemoembolization; TFS, transplant-free survival.

In the derivation cohort, 27% of patients had very early or early-stage HCC (0/A), 22.02% had intermediate-stage (B), 45.87% had advanced-stage (C), and 5.05% had terminal-stage (D). By contrast, in the validation cohort the proportions were 43.75% (0/A), 25.00% (B), 5.21% (C), and 26.04% (D), respectively. Moreover, 22.07% of the derivation cohort had extrahepatic metastases at the time of presentation.

The median age of the derivation cohort and the validation cohort was significantly different (*P*<.001) (Table 1). The admission bilirubin (*P*<.001), and day 7 bilirubin (*P*=.006) were significantly higher in the derivation cohort than those in the validation cohort. The time to death from admission was longer in the validation cohort (45 vs 79 days; *P*=.010). The distribution of BCLC staging in the derivation cohort was significantly different to the validation cohort (*P*<.001). The validation set had larger average tumor sizes than the derivation set (5.78±4.53 vs 4.15±3.34; *P*=.014).

On univariable analysis, the following parameters were identified as significant for predicting 90-day mortality: sodium (*P*=.001), creatinine (*P*=.002), bilirubin (*P*<.001), albumin (*P*=.021), and INR (*P*=.025); day 7 sodium (*P*=.002), bilirubin (*P*<.001), and change in bilirubin (*P*=.002) (Supplemental Table 2, available online at http://www.mcpiqojournal.org). In multivariable analysis bilirubin (odds ratio [OR], 1.02; 95% CI, 1.00-1.03; *P*=.009) and change in bilirubin (OR, 1.01; 95% CI, 1.00-1.02; *P*=.018) remained significant for predicting 90-day mortality.

**Figure 1:**
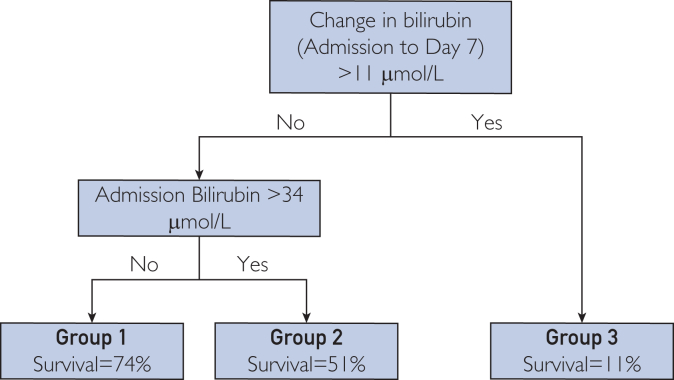
The HCC-AD model for prediction of 90-day mortality in patients with hepatocellular carcinoma experiencing an acute decompensatory event of cirrhosis.

Bilirubin and change in bilirubin were used in CART analysis to develop the HCC-AD model (Figure 1). Cutoff values of change in bilirubin <11 μmol/L and serum bilirubin at admission of <34 μmol/L were selected as most discriminatory. Patients in the validation cohort were stratified into one of 3 groups (groups A–C) based on the HCC-AD model. Patients in group A had a predicted mortality of 26% compared with an observed morality of 21.40%. Patients in group B had predicted and observed mortalities of 49% and 61.90%, respectively, whereas those in group C had 89% and 87.50% (Supplemental Figure 6, available online at http://www.mcpiqojournal.org).

The area under receiver operating characteristic curve of the HCC-AD in the validation cohort was 0.79 (95% CI, 0.69-0.90) as compared to 0.81 (95% CI, 0.72-0.89) and 0.73 (95% CI, 0.64-0.82) for the MELD score and ALBI score at admission respectively (Supplemental Figure 7, available online at http://www.mcpiqojournal.org). The HCC-AD showed higher overall discrimination (Harrell C=0.73) than the ALBI (Harrell C=0.68; *P*=.36) and Child-Pugh (Harrell C=0.70; *P*=.73) (Table 2). It also showed noninferior discrimination to the MELD 3.0 score at admission (Harrell C=0.73; *P*=.67). On multivariate analysis the predictive capacity of the HCC-AD score (*P*=.001) was not affected when adjusting for the CLIF-C AD score (*P*=.14).

**Table 2:** Discrimination of HCC-AD, MELD 3.0, Child-Pugh, and ALBI Scores

Score	HCC-AD	MELD 3.0 score	Child-Pugh score	ALBI score
Harrell C	0.729	0.733	0.701	0.684
*P* value vs HCC-AD	—	.669	.732	.360
95% CI vs HCC-AD	—	−0.181 to 0.117	−0.100 to 0.142	−0.078 to 0.211

AD, acute decompensation; ALBI, albumin-bilirubin ratio; BCLC, Barcelona Clinic Liver Cancer; HCC, hepatocellular carcinoma; MELD, model for end-stage liver disease.

**Figure 2:**
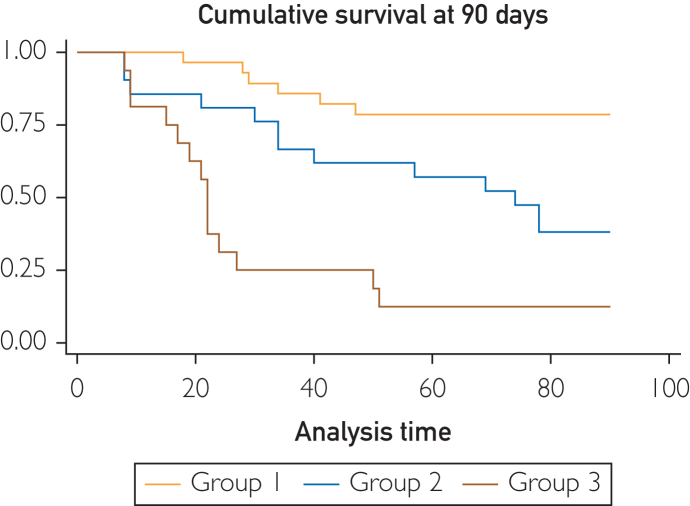
Cumulative survival at 90 days following admission in validation cohort as stratified by HCC-AD.

On Kaplan-Meier analysis, survival distribution between HCC-AD cohorts was significantly different between all groups (1 and 2 [χ^2^=7.74; *P*=.005]; 1 and 3 [χ^2^=26.53; *P*<.001]; 2 and 3 [χ^2^=6.88; *P*=.009]) (Figure 2). Based on visual inspection, the new predictive model was well calibrated (Supplemental Figure 8, available online at http://www.mcpiqojournal.org).

Vascular invasion (*P*=.003), extrahepatic metastases (*P*=.046), and BCLC stage (*P*<.001) were all independent predictors of 90-day mortality in the validation cohort on univariate analysis. However, multivariate regression analysis determined that BCLC stage (*P*=.67) did not show a relationship with overall survival when adjusted for the HCC-AD model (Supplemental Table 3, available online at http://www.mcpiqojournal.org). Similarly, the presence of vascular invasion (*P*=.99) or extrahepatic metastases (*P*=.49) did not affect the capacity of the HCC-AD score to predict 90-day mortality. The Milan Criteria (*P*=.92) and Metroticket calculator for HCC survival (*P*=.83) did not influence the HCC-AD scores predictive capacity. There was no impact of previous treatment on the predictive capacity of the HCC-AD score, with previous resection (*P*=.95), ablation (*P*=.34), transarterial chemoembolization (*P*=.079), or systemic chemotherapy (*P*=.59) having no significant impact (Supplemental Table 4, available online at http://www.mcpiqojournal.org).

The HCC-AD score remained predictive of 90-day mortality in patients with BCLC B, C, and D HCC (*P*<.001; *P*<.001; and *P*=.010, respectively) (Table 3). For patients with intermediate-stage HCC, a 1-point increased in HCC-AD score conferred a 11.44-fold increase in risk of 90-day mortality, whereas there was a 7.03-fold and 10.54-fold increased risk for patients with advanced and terminal HCC. Although the MELD 3.0 and Child-Pugh scores were prognostic for 90-day mortality in patients with intermediate (*P*<.001 and *P*<.001, respectively) and advanced (*P*=.002 and *P*=.001, respectively) HCC, they did not account for terminal-stage HCC (*P*=.058 and *P*=.11, respectively). No score was predictive in patients with BCLC A HCC (HCC-AD, *P*=.44; MELD 3.0, *P*=.57; Child-Pugh, *P*=.70). Day 7 MELD 3.0 score was predictive of 90-day mortality in BCLC B (*P*<.001) and BCLC C (*P*=.001) patients, whereas the 7-day change in MELD 3.0 score was not predictive. When adjusted for the HCC-AD score, day 7 MELD 3.0 score did not remain significant in patients with BCLC C (*P*=.10).

**Table 3:** Subgroup Analysis of HCC-AD, MELD 3.0, and Child-Pugh Scores in Each BCLC Stage of HCC

BCLC	HCC-AD			MELD 3.0			Child-Pugh score		
	Coefficient (95% CI)	Odds ratio (95% CI)	*P*	Coefficient (95% CI)	Odds ratio (95% CI)	*P*	Coefficient (95% CI)	Odds ratio (95% CI)	*P*
0/A	0.34 (−0.52 to 1.19)	1.40 (0.60-3.29)	.441	0.02 (−0.05 to 0.09)	1.02 (0.95-1.09)	.565	−0.05 (−0.29 to 0.20)	0.95 (0.75-1.22)	.704
B	2.43 (1.22-3.65)	11.44 (3.40-38.53)	<.001	0.11 (0.05-0.18)	1.13 (1.05-1.20)	<.001	0.70 (0.35-1.05)	2.02 (1.42-2.86)	<.001
C	1.95 (1.02-2.88)	7.03 (2.76-17.88)	<.001	0.11 (0.04-0.18)	1.12 (1.04-1.20)	.002	0.46 (0.19-0.74)	1.60 (1.21-2.10)	.001
D	2.56 (0.57-4.14)	10.54 (1.77-62.66)	.010	0.14 (−0.00 to 0.28)	1.15 (1.00-1.33)	.058	0.46 (−0.10 to 1.03)	1.59 (0.91-2.79)	.106

AD, acute decompensation; ALBI, albumin-bilirubin ratio; BCLC, Barcelona Clinic Liver Cancer; HCC, hepatocellular carcinoma; MELD, model for end-stage liver disease.

Firth’s penalized cox regression with an interaction term was used for gender to minimize the bias of the small number of female participants. The interaction between the HCC-AD score and gender did not significantly modify the capacity of the HCC-AD score to predict 90-day mortality (*P*=.74). The HCC-AD score remained predictive for patients with disease related to alcohol (*P*<.001), hepatitis B virus (*P*<.001), HCV (*P*<.001), and metabolic dysfunction–associated liver disease (*P*<.001) (Supplemental Table 4, available online at http://www.mcpiqojournal.org). In patients with a decompensatory event that was secondary to progression of HCC, the HCC-AD score remained significant for predicting 90-day mortality (*P*=.003) (Supplemental Table 5, available online at http://www.mcpiqojournal.org).

## Discussion

We developed a dynamic score (the HCC-AD score) that predicts 90-day mortality for patients with synchronous HCC and acutely decompensated liver disease. Patients with HCC secondary to advanced liver disease are well known to health services, making objective decision making on treatment futility difficult. Although the MELD, Child-Pugh, and ALBI scores are commonly used in prognostication, they do not account for changes in clinical status during an admission. The Cancer of the Liver Italian Program score also does not account for acute changes during the admission.^12^ We show that 7-day change in bilirubin is superior to other laboratory parameters and scores in predicting 90-day TFS in patients with HCC experiencing an acute decompensatory event (Supplemental Figures 2-5, available online at http://www.mcpiqojournal.org). Furthermore, a simple predictive model (HCC-AD) incorporating bilirubin and its 7-day change is a superior prognostic marker of 90-day mortality. Subgroup analysis confirms that it is valid in intermediate-stage, advanced-stage, and terminal-stage HCC, whereas decompensatory events precipitated by tumor progression are also accounted for. Moreover, it is predictive independent of liver disease etiology and gender.

Acute decompensations of advanced liver disease are predictive of mortality in patients with HCC.^4^ The predictive capacity of HCC-AD was noninferior to current scores and remained an independent predictor of mortality when adjusting for BCLC class. Perhaps, the most relevant cohort to identify futility of intervention, BCLC D, is only accounted for by the HCC-AD score, with the MELD and Child-Pugh scores not remaining independently predictive. Thus, HCC-AD enables early identification of patients that are not candidates for ongoing invasive therapy. Further, risk stratification is informed by a patient’s evolving condition, with dynamic scores historically demonstrating superior ability to identify mortality risk.^16^, ^17^, ^18^ Although the MELD score offers good prognostic information at admission it does not account for changes following any therapeutic intervention and/or progression of decompensatory events.^19^ The HCC-AD score also outperforms day 7 MELD 3.0 and 7-day change in MELD 3.0 in predicting 90-day TFS. Bilirubin kinetics are superior to the MELD, Child-Pugh, and ALBI scores in predicting 90-day survival (Supplemental Appendix 1, available online at http://www.mcpiqojournal.org). The cutoff value of a change of less than 11 μmol/L had a specificity of 93.24% and positive likelihood ratio of 7.30. The HCC-AD minimizes false-positive results, which was optimal to identify futility of ongoing treatment, helping to capture patients who would otherwise continue with invasive treatment.

Studies have shown that patients facing end of life care still undergo high levels of nonbeneficial treatments—compromising quality of life and putting additional stress on families, treating staff and the health care system.^20^^,^^21^ Thus, the HCC-AD can be useful to predict the futility of ongoing therapy and for improving the quality of life of patients. We showed that the HCC-AD score also accounts for the most vulnerable population, patients with terminal-stage HCC, whereas others do not. Dynamic models such as the HCC-AD can also be used for appropriate postdischarge planning,^22^ providing prognostic information to patients and enabling objective measures for considered decision making whereby quality of life and psychological well-being are prioritized.^23^ The use of dynamic modeling is justified when adjusting for an independent acuity score (CLIF-C AD score) on multivariate analysis. Failure of the CLIF-C AD score to reach statistical significance indicates that patient characteristics on admission alone are not capable of determining 90-day TFS.

The liver is inextricably involved in the metabolism of bilirubin, with increased serum levels often indicating severe hepatic dysfunction in cirrhosis.^24^ It is therefore unsurprising that both serum bilirubin and 7-day change are significantly associated with 90-day mortality. Decreased hepatic clearance (owing to low glucuronyl conjugation and biliary excretion) complicated by increased de novo production through hemolysis (induced by red blood cell structural abnormalities secondary to portal hypertension and subsequent splenomegaly) contribute.^25^ Understandably, failure of these processes to correct with supportive therapy, characterized by an increased day 7 bilirubin, represents ongoing progression and deterioration of liver disease. Despite development as a score for patients with HCC, there are no tumor-specific features incorporated into the HCC-AD score. A study of patients with treated BCLC A HCV-related HCC showed that hepatic decompensation was more prognostic of short-term survival when compared with development of HCC.^26^ Therefore, it is reasonable to expect that a score may prioritize severity of hepatic decompensation over tumor factors. Vascular invasion and extrahepatic metastases were predictive of 90-day TFS on univariate analysis but did not remain when adjusting for the HCC-AD score. Change in bilirubin may account for these phenomena. Tumor progression, invasion of hepatic parenchyma and bile duct thrombi are all associated with mortality in HCC.^27^ The literature shows that the prognosis of patients with HCC infiltration/obstruction-related jaundice is better than those with raised bilirubin secondary to an acute decompensation.^28^^,^^29^ This is because early surgical intervention can result in prolonged overall survival.^29^^,^^30^ It can therefore be argued that the new dynamic score would better account for this subset of patients because the relief of obstruction would result in significant 7-day decreases in serum bilirubin accurately placing these patients in the low-risk group. We showed that the HCC-AD score remained predictive for survival when the precipitant for decompensation was progression of primary tumor. HCC-AD would better account for these patients than current static scores. In our study, despite the admission serum bilirubin being significantly different in the derivation and validation cohorts, the HCC-AD was able to capture survival in both. This suggests that perhaps there is a threshold admission bilirubin value (a surrogate for hepatic reserve) above which increased levels do not negatively impact survival.

This study has limitations. First, the study used electronic health records from a single health district, consequently some patients may have been lost to follow up. Second, because the study is retrospective in nature, there is the possibility of misclassification and incorrect recording of variables. Based on our data, we suggest that a prospective trial should be conducted to validate the present findings. Owing to the retrospective nature, in-hospital interventions and discharge destinations could not reliably recorded. It is unclear how these may influence the predictive capacity of the HCC-AD score. Third, despite being a score designed for HCC, there are no tumor-specific features incorporated into the HCC-AD score. On regression analysis no tumor-specific features were predictive of 90-day TFS when adjusting for the HCC-AD score. However, on subgroup analysis patients who presented secondary to tumor progression were accounted for by the score indicating its clinical utility. Additionally, the HCC cohort was predominantly male, perhaps limiting its predictive capacity among females. Although regression analysis showed no effect of gender on HCC-AD prediction, further validation using a cohort with better female representation would be useful. Finally, the HCC-AD score does not account for patients with early-stage HCC. It is important to acknowledge that as a tool for assessing futility of ongoing therapy the HCC-AD score would rarely be relevant for patients early in their disease course. Further studies evaluating predictive models for early-stage HCC are indicated.

## Conclusion

We identified that 7-day change in bilirubin is the best dynamic predictor of 90-day survival in patients with HCC experiencing an acute decompensation of cirrhosis. A newly derived HCC-AD score is an objective decision-support tool that can be used to determine prognosis and futility of ongoing management. Subgroup analysis confirmed HCC-AD is prognostic for intermediate-stage, advanced-stage, and terminal-stage HCC, which is not reflected by current scores. Further evaluation of mortality in early-stage HCC is necessary.

## Potential Competing Interests

The authors report no competing interests.

## Ethics Statement

This study was performed in compliance with Australian legislation and the Declaration of Helsinki regarding ethics principles for medical research involving human subjects. The study was approved by the ethics committee of Western Sydney Local Health District (HREA 2021/ETH00149). A waiver of consent was applied due to the retrospective nature of this study based on criteria outlined in the Australian National Statement on Ethical Conduct in Human Research.

## References

[1] Tandon P., Garcia-Tsao G. (2009). Prognostic indicators in hepatocellular carcinoma: a systematic review of 72 studies. Liver Int.

[2] Bosch F.X., Ribes J., Cléries R., Díaz M. (2005). Epidemiology of hepatocellular carcinoma. Clin Liver Dis.

[3] Kulik L., El-Serag H.B. (2019). Epidemiology and management of hepatocellular carcinoma. Gastroenterology.

[4] Kondo T., Koroki K., Kanzaki H. (2022). Impact of acute decompensation on the prognosis of patients with hepatocellular carcinoma. PLoS One.

[5] Johnson P.J., Berhane S., Kagebayashi C. (2015). Assessment of liver function in patients with hepatocellular carcinoma: a new evidence-based approach—the ALBI grade. J Clin Oncol.

[6] Calderon-Martinez E., Landazuri-Navas S., Vilchez E. (2023). Prognostic scores and survival rates by etiology of hepatocellular carcinoma: a review. J Clin Med Res.

[7] Demirtas C.O., D’Alessio A., Rimassa L., Sharma R., Pinato D.J. (2021). ALBI grade: evidence for an improved model for liver functional estimation in patients with hepatocellular carcinoma. JHEP Rep.

[8] Liu P.H., Hsu C.Y., Hsia C.Y. (2016). Prognosis of hepatocellular carcinoma: assessment of eleven staging systems. J Hepatol.

[9] Hiraoka A., Michitaka K., Kumada T. (2017). Validation and potential of albumin-bilirubin grade and prognostication in a nationwide survey of 46,681 hepatocellular carcinoma patients in japan: the need for a more detailed evaluation of hepatic function. Liver Cancer.

[10] Sarkar J., Deleon T., Wong L.L. (2017). MELD score and AST-to-platelet ratio index predict long-term survival in patients with a small hepatocellular carcinoma following non-transplant therapies: a pilot study. Hepatoma Res.

[11] Cucchetti A., Ercolani G., Vivarelli M. (2006). Impact of model for end-stage liver disease (MELD) score on prognosis after hepatectomy for hepatocellular carcinoma on cirrhosis. Liver Transpl.

[12] Maida M., Orlando E., Cammà C., Cabibbo G. (2014). Staging systems of hepatocellular carcinoma: a review of literature. World J Gastroenterol.

[13] Louvet A., Naveau S., Abdelnour M. (2007). The Lille model: a new tool for therapeutic strategy in patients with severe alcoholic hepatitis treated with steroids. Hepatology.

[14] Louvet A., Labreuche J., Artru F. (2015). Combining data from liver disease scoring systems better predicts outcomes of patients with alcoholic hepatitis. Gastroenterology.

[15] Qiao L., Tan W., Wang X. (2021). Different effects of total bilirubin on 90-day mortality in hospitalized patients with cirrhosis and advanced fibrosis: a quantitative analysis. Front Med (Lausanne).

[16] Amin S.T., Morrow D.A., Braunwald E. (2013). Dynamic TIMI risk score for STEMI. J Am Heart Assoc.

[17] Wilbaux M., Hénin E., Oza A. (2014). Dynamic modeling in ovarian cancer: an original approach linking early changes in modeled longitudinal CA-125 kinetics and survival to help decisions in early drug development. Gynecol Oncol.

[18] Esteban C., Staeck O., Yang Y., Tresp V. (Published online November 17, 2016).

[19] Peeraphatdit T., Naksuk N., Thongprayoon C. (2015). Prognostic value of model for end-stage liver disease score measurements on a daily basis in critically ill patients with cirrhosis. Mayo Clin Proc.

[20] Cardona-Morrell M., Kim J., Turner R.M., Anstey M., Mitchell I.A., Hillman K. (2016). Non-beneficial treatments in hospital at the end of life: a systematic review on extent of the problem. Int J Qual Health Care.

[21] Aghabarary M., Dehghan Nayeri N. (2016). Medical futility and its challenges: a review study. J Med Ethics Hist Med.

[22] Califf R.M., Pieper K.S., Lee K.L. (2000). Prediction of 1-year survival after thrombolysis for acute myocardial infarction in the global utilization of streptokinase and TPA for occluded coronary arteries trial. Circulation.

[23] Chu C., White N., Stone P. (2019). Prognostication in palliative care. Clin Med (Lond).

[24] Guerra Ruiz A.R., Crespo J., López Martínez R.M. (2021). Measurement and clinical usefulness of bilirubin in liver disease. Adv Lab Med.

[25] Ohkubo A. (1994). [Bilirubin metabolism in liver cirrhosis]. Nihon Rinsho.

[26] Cabibbo G., Petta S., Barbara M. (2017). Hepatic decompensation is the major driver of death in HCV-infected cirrhotic patients with successfully treated early hepatocellular carcinoma. J Hepatol.

[27] Mesci A., Gurer S., Guzel G., Demirbakan K. (2008). Obstructive jaundice caused by hepatocellular carcinoma with bile duct tumor thrombi: a case report. Eurasian J Med.

[28] Peng S.Y., Wang J.W., Liu Y.B. (2004). Surgical intervention for obstructive jaundice due to biliary tumor thrombus in hepatocellular carcinoma. World J Surg.

[29] Peng B.G., Liang L.J., Li S.Q., Zhou F., Hua Y.P., Luo S.M. (2005). Surgical treatment of hepatocellular carcinoma with bile duct tumor thrombi. World J Gastroenterol.

[30] Elshimi E., Morad W. (2020). Cost analysis of biliary drainage using metal versus plastic stents in hepatocellular carcinoma patients with obstructive jaundice. Gastrointest Tumors.

