# Supplementary material for: Dynamic Changes in Bilirubin Predict 90-Day Mortality in Patients With Hepatocellular Carcinoma and Acute Decompensations of Cirrhosis: The HCC-AD Score
Source: Mayo Clin Proc Innov Qual Outcomes. 2025 Sep 12;9(5):100661. doi: 10.1016/j.mayocpiqo.2025.100661 (PMC12541609; doi:10.1016/j.mayocpiqo.2025.100661)

Dynamic changes in bilirubin predict 90-day mortality in hepatocellular carcinoma patients with acute decompensations of cirrhosis: the HCC-AD score

Supplementary Material

Table of contents Page

1. Patient selection 2
2. Baseline characteristics of derivation cohort. 3
3. Univariate and multivariate analyses of baseline variables for predicting 90-day mortality 5
4. 7-day change in bilirubin is most predictive of 90-day mortality 6
5. Evaluation of tumor specific characteristic on predicting 90-day mortality when adjusted for HCC-AD score 8
6. Impact of tumour related factors and previous therapy on HCC-AD’s performance. 9
7. Impact of aetiology on predictive capacity of HCC-AD score 10
8. Subgroup analysis of HCC-AD score for patient depending on the precipitant of decompensation. 11
9. 90-day mortality as predicted by 7-day change in Bilirubin. 12
10. 90-day mortality as predicted by 7-day change in MELD Score. 13
11. 90-day mortality as predicted by 7-day change in ALBI Score. 14
12. 90-day mortality as predicted by 7-day change in Child Pugh Score. 15
13. Observed and predicted mortality in validation group based on HCC-AD group 16
14. AUROC of the various scores. 17
15. Observed vs predicted survival based on HCC-AD. 18

1. **Patient selection**

Figure S1: Patient selection

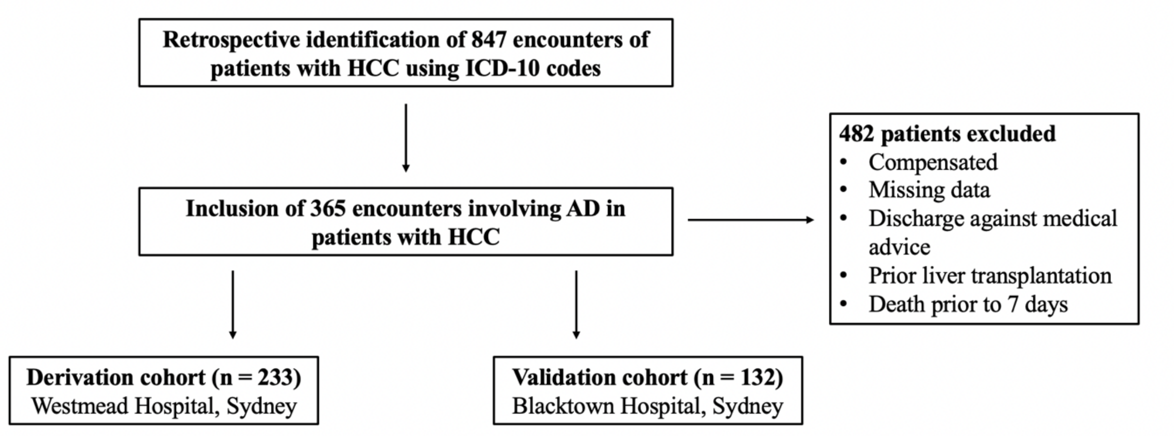

2. **Baseline characteristics of derivation cohort**

Table S1: Baseline Characteristics among the surviving and non-surviving patients in the derivation cohort

	**Survival Group (n= 124)**	**Deceased Group (n=109)**	**p-Value**
**Age,** (mean, IQR)	66.00 (11.0)	65.00 (10.00)	*0.060*
**Male sex,** (n, %)	93 (85.32)	91(73.39)	0.11
**Aetiology of liver disease,** (n, %)			
EtOH	25 (22.94)	33 (26.61)	0.075
HCV	58 (53.21)	42 (33.87)	0.21
HBV	18 (16.51)	26 (20.97)	0.069
MASH	27 (24.77)	14 (11.29)	0.074
**Sign of AD at inclusion,** (n, %)			
Ascites	63 (50.80)	67 (54.03)	0.10
Hepatic Encephalopathy	55 (44.35)	44 (35.48)	0.60
Variceal Bleeding	17 (13.71)	13 (10.48)	0.69
**Admission Laboratory Parameters**			
Sodium (mmol/L)	136.00 (6.0)	133.00 (7.0)	*0.0001*
Creatinine (mmol/L)	79.00 (49.0)	92.50 (87.0)	*0.030*
Bilirubin (umol/L)	33.00 (44.50)	67.50 (67.0)	*<0.0001*
Albumin (mmol/L)	27.00 (7.0)	25.00 (7.50)	*0.0003*
INR	1.30 (0.35)	1.50 (0.65)	*0.0088*
**Day 7 Laboratory Parameters**			
Sodium (mmol/L)	136.00 (6.0)	134.00 (6.0)	*0.0006*
Creatinine (mmol/L)	76.00 (46.0)	85.00 (71.0)	0.32
Bilirubin (umol/L)	31.00 (50.0)	82.00 (102.5)	*<0.001*
Albumin (mmol/L)	28.00 (7.0)	27.00 (9.0)	0.33
INR	1.40 (0.40)	1.55 (0.50)	*0.018*
**Change over first 7-days**			
Sodium (mmol/L)	0.00 (5.0)	0.00 (6.0)	0.19
Creatinine (mmol/L)	-3.00 (24.0)	-5.00 (45.0)	0.29
Bilirubin (umol/L)	-4.00 (15.0)	10.00 (43.0)	*0.0019*
Albumin (mmol/L)	1.00 (5.0)	2.00 (9.0)	0.42
INR	0.00 (0.20)	0.00 (0.30)	0.099
**ALBI Score,** (mean ± SD)			
Admission	-1.34 (0.65)	-0.88 (0.90)	0.43
Day 7	-1.44 (0.66)	-1.10 (0.88)	0.54

7-day change	-0.12 (0.45)	-0.07 (0.70)	0.45
**MELD 3.0 Score,** (mean ± SD)			
Admission	16.84 (10.20)	22.52 (9.11)	*< 0.001*
Day 7	15.87 (10.53)	22.31 (8.73)	*< 0.001*
7-day change	0.93 (4.56)	0.03 (3.25)	0.16
**Child Pugh Score,** (mean ± SD)			
Admission	8.00 (3.0)	10.00 (3.0)	*< 0.001*
Day 7	8.00 (2.0)	9.00 (3.0)	*< 0.001*
7-day change	0.00 (1.0)	0.00 (1.0)	0.57
**Decompensation due to HCC progression,** (n, %)	18 (14.75)	24 (23.08)	0.11
**BCLC Class,** (n, %)			*<0.001*
A/0	42 (35.59)	17 (17.00)	
B	31 (26.27)	17 (17.00)	
C	45 (38.14)	55 (55.00)	
D	0 (0.00)	11 (11.00)	
**Radiological Characteristic**			
Tumour Number			0.53
1	37 (31.90)	35 (38.46)	
2	23 (19.83)	14 (15.38)	
3	14 (12.07)	7 (7.69)	
4 or more	42 (36.21)	35 (38.46)	
Largest tumour size	3.00 (3.50)	3.40 (4.50)	0.55
Portal Hypertension	117 (100.00)	98 (98.99)	0.28
Tumour related PVT	38 (33.63)	56 (58.95)	*< 0.001*
Extra-hepatic metastases	16 (13.68)	31 (32.29)	*0.001*
**Previous Therapy**			
Resection	5 (4.03)	3 (2.75)	0.59
Microwave Ablation	23 (18.55)	20 (18.35)	0.97
TACE	34 (27.42)	36 (33.03)	0.35
SIRT	5 (4.03)	11 (10.09)	0.068
SBRT	1 (0.81)	5 (4.59)	0.069
Chemotherapy	27 (21.26)	39 (35.78)	*0.018*
Immunotherapy	4 (3.23)	1 (0.92)	0.23

3. Univariate and multivariate analyses of baseline variables for predicting 90-day mortality

Table S2: Univariate and multivariate analyses of baseline variables for predicting 90-day mortality.

Univariate Analysis

	Regression Coefficient	Standard Error	Unadjusted OR (95% CI)	p Value
Admission Sodium	-0.094	0.027	0.91 (0.86, 0.96)	0.001
Day 7 Sodium	-0.11	0.033	0.90 (0.84, 0.96)	0.002
Admission Potassium	0.72	0.18	2.05 (1.44, 2.92)	>0.001
Change in Potassium	-0.77	0.22	0.46 (0.30, 0.72)	0.001
Admission Creatinine	0.0078	0.0026	1.01 (1.00, 1.01)	0.002
Admission Bilirubin	0.012	0.0031	1.01 (1.01, 1.02)	>0.001
Day 7 Bilirubin	0.011	0.0032	1.012 (1.01, 1.02)	>0.001
Change in Bilirubin	0.018	0.0058	1.019 (1.01, 1.03)	0.002
Admission Albumin	-0.052	0.021	0.95 (0.91, 0.99)	0.021
Admission INR	0.67	0.30	1.96 (1.09, 3.52)	0.025

Multivariate Analysis

	Regression Coefficient	Standard Error	Adjusted OR (95% CI)	p Value
Admission Bilirubin	0.016	0.005	1.02 (1.00, 1.03)	0.009
Change in Bilirubin	0.01	0.004	1.01 (1.00, 1.02)	0.018

4. 7-day change in bilirubin is most predictive of 90-day mortality

Bilirubin has long been appreciated as a measure of underlying hepatic dysfunction, with increases in serum bilirubin known to be an independent predictor of 90-day mortality in patients with cirrhosis (1, 2). The predictive role of dynamic changes in bilirubin is well established for patients with alcoholic hepatitis (3-5) and those with acute on chronic liver failure (6). However, there has been no investigation into dynamic changes in bilirubin in patients with HCC. Similarly, dynamic changes in the MELD score are predictive of mortality however they have not been investigated in HCC cohorts (7, 8). The utility of the ALBI score in those patients with HCC experiencing an acute decompensatory event has not been investigated (9, 10).

Performance of 7-day change in predicting 90-day mortality.

Sensitivity, specificity, positive predictive value (PPV), and negative predictive value (NPV) were calculated for the 7-day change in bilirubin, MELD 3.0 score, Child Pugh score, and ALBI score in predicting 90-day survival. Differences between groups were considered statistically significant when p was < 0.05.

Prediction of mortality based on serum bilirubin not changing or increasing at 7 days yielded a sensitivity of 67.53% (95% CI 60.06 – 75.00) and specificity of 67.57% (95% CI 60.10 – 75.03) (Figure S2). Larger increases in bilirubin yielded a higher specificity and positive predictive value for 90-day mortality, with a serum bilirubin increase of ≥10 umol/L resulting in a sensitivity of 50.65% (95% CI 42.68 – 58.62) and specificity of 91.89% (87.54 – 96.25) for 90-day mortality. This also resulted in a strong positive predictive value of 86.67% (95% CI 81.24 – 92.09). Comparatively, if the MELD 3.0 score remained the same or increased, a sensitivity of 50.00% (95% CI 41.20 – 58.80) and specificity of 35.71 (95% CI 27.28 – 44.15) was yielded (Figure S3), whilst the ALBI score returned a poor sensitivity of 35.06% (95% CI 27.45 – 42.68) and specificity of 62.16% (54.43 – 69.90) (Figure S4). The Child Pugh score had a sensitivity of 82.61% (95% CI 75.96 – 89.25) but a poor specificity of 14.29% (95% CI 8.15 – 20.42) (Figure S5). In the derivation cohort the Area under to ROC curve for change in bilirubin was 0.727 compared with 0.441 ($p < 0.001$), 0.470 ($p < 0.001$) and 0.499 ($p < 0.001$) for changes in the MELD 3.0, Child Pugh and ALBI scores respectfully.

We completed logistic regression analysis to evaluate the capacity of bilirubin to predict 90-day mortality. For every 10 umol/L increase in bilirubin at 7 days there is a 1.5-time higher risk of 90-day mortality, underpinning the importance of dynamic changes in bilirubin. Receiver operating characteristic (ROC) curve analysis with Youden's index to identify the optimal cut off for bilirubin. A cut off of 4.5 was found to have a sensitivity of 61% and specificity of 84%. However, as the intention of the HCC-AD score was to develop an index of futility of ongoing intervention, CART analysis selected a cut off less than 11 as having a suitably high specificity and positive likelihood value to minimise the chance of false positives. Prioritising selecting those patients that are most vulnerable to futile investigations is the intention of the HCC-AD score.

In advanced liver disease, multiple pathophysiological processes result in raised bilirubin with increased concentrations widely accepted as a late event (11). Hepatic clearance of bilirubin becomes impaired due to decreased glucuronyl conjugation and biliary excretion (12). Portal hypertension and subsequent splenomegaly also result in structural abnormalities of red blood cells, causing higher levels of hemolysis which contributes to high production of bilirubin (13). Understandably, failure of these processes to correct with supportive therapy, characterized by an increased day 7 bilirubin, represents ongoing progression and deterioration of liver disease. In this context, is not surprising that 7-day change in bilirubin is more discriminatory than other pathological variables, and current score such as the MELD 3.0, Child Pugh and ALBI.

References

1. Qiao L, Tan W, Wang X, Zheng X, Huang Y, Li B, et al. Different Effects of Total Bilirubin on 90-Day Mortality in Hospitalized Patients With Cirrhosis and Advanced Fibrosis: A Quantitative Analysis. Front Med (Lausanne). 2021;8:704452.
2. Tandon P, Garcia-Tsao G. Prognostic indicators in hepatocellular carcinoma: a systematic review of 72 studies. Liver Int. 2009;29(4):502-10.
3. Mathurin P, Abdelnour M, Ramond MJ, Carbonell N, Fartoux L, Serfaty L, et al. Early change in bilirubin levels is an important prognostic factor in severe alcoholic hepatitis treated with prednisolone. Hepatology. 2003;38(6):1363-9.
4. Parker R, Cabezas J, Altamirano J, Arab JP, Ventura-Cots M, Sinha A, et al. Trajectory of Serum Bilirubin Predicts Spontaneous Recovery in a Real-World Cohort of Patients With Alcoholic Hepatitis. Clin Gastroenterol Hepatol. 2022;20(2):e289-e97.
5. Lee M, Kim W, Choi Y, Kim S, Kim D, Yu SJ, et al. Spontaneous Evolution in Bilirubin Levels Predicts Liver-Related Mortality in Patients with Alcoholic Hepatitis. PLoS ONE. 2014;9(7):e100870.
6. Lin W, Zhang J, Liu X, Liu H, He J, Li M, et al. A Dynamic Model for Predicting Outcome in Patients with HBV Related Acute-On-Chronic Liver Failure. Annals of Hepatology. 2018;17(3):392-402.
7. Kumar R, Krishnamoorthy TL, Tan HK, Lui HF, Chow WC. Change in model for end-stage liver disease score at two weeks, as an indicator of mortality or liver transplantation at 60 days in acute-on-chronic liver failure. Gastroenterol Rep (Oxf). 2015;3(2):122-7.
8. Zheng YB, Huang ZL, Wu ZB, Zhang M, Gu YR, Su YJ, et al. Dynamic changes of clinical features that predict the prognosis of acute-on-chronic hepatitis B liver failure: a retrospective cohort study. Int J Med Sci. 2013;10(12):1658-64.
9. Lin PT, Teng W, Jeng WJ, Chen WT, Hsieh YC, Huang CH, et al. Dynamic Change of Albumin-Bilirubin Score Is Good Predictive Parameter for Prognosis in Chronic Hepatitis C-hepatocellular Carcinoma Patients Receiving Transarterial Chemoembolization. Diagnostics (Basel). 2022;12(3).
10. Ananchuensook P, Sriphoosanaphan S, Suksawatamnauy S, Siripon N, Pinjaroen N, Geratikornsupuk N, et al. Validation and prognostic value of EZ-ALBI score in patients with intermediate-stage hepatocellular carcinoma treated with trans-arterial chemoembolization. BMC Gastroenterology. 2022;22(1).
11. Fevery J. Bilirubin in clinical practice: a review. Liver International. 2008;28(5):592-605.
12. Ohkubo A. [Bilirubin metabolism in liver cirrhosis]. Nihon Rinsho. 1994;52(1):138-44.
13. Morse EE. Mechanisms of hemolysis in liver disease. Ann Clin Lab Sci. 1990;20(3):169-74.

5. **Evaluation of tumor specific characteristic on predicting 90-day mortality when adjusted for HCC-AD score**

Table S3: Univariate and multivariate analyses of tumor specific features for predicting 90-day mortality.

Univariate Analysis				
	**Regression Coefficient**	**Standard Error**	**Unadjusted OR (95% CI)**	**p Value**
Tumor Number	0.15	0.14	1.17 (0.88, 1.55)	0.288
Largest Tumor	-0.03	0.06	0.97 (0.86, 1.09)	0.587
Vascular Invasion	1.26	0.43	3.52 (1.53, 8.13)	*0.003*
Extra-hepatic Metastases	1.66	0.83	5.25 (1.03, 26.72)	*0.046*
BCLC	0.83	0.19	2.30 (1.58, 3.34)	*<0.001*

Multivariate Analysis				
	**Regression Coefficient**	**Standard Error**	**Adjusted OR (95% CI)**	**p Value**
HCC-AD Score	1.57	0.55	4.80 (1.62, 14.23)	*0.005*
Vascular Invasion	-0.00	0.74	1.00 (0.23, 4.23)	0.99
Extra-hepatic Metastases	1.22	1.77	3.40 (0.11, 108.73)	0.49
BCLC	0.20	0.55	1.22 (0.50, 2.99)	0.67

6. **Impact of tumour related factors and previous therapy on HCC-AD's performance.**

Table S3:

a) **Impact of BCLC on HCC-AD's performance.**

	**Coefficient**	**95% CI**	**p Value**
HCC-AD	1.54	0.65, 2.42	*0.001*
BCLC	0.29	-0.30, 0.89	0.335

b) **Impact of tumour related thrombus on HCC-AD's performance.**

	**Coefficient**	**95% CI**	**p Value**
HCC-AD	1.60	0.69, 2.50	*0.001*
Tumour associated thrombus	0.38	-0.91, 1.68	0.562

c) **Impact of extra-hepatic metastases on HCC-AD's performance.**

	**Coefficient**	**95% CI**	**p Value**
HCC-AD	1.71	0.81, 2.61	*< 0.001*
Extra-hepatic Metastases	1.70	-1.15, 4.53	0.243

d) **Impact of previous resection on HCC-AD's performance.**

	**Coefficient**	**95% CI**	**p Value**
HCC-AD	1.66	0.82, 2.50	*< 0.001*
Previous Resection	-0.08	-2.75, 2.59	0.954

e) **Impact of microwave ablation on HCC-AD's performance.**

	**Coefficient**	**95% CI**	**p Value**
HCC-AD	1.61	0.77, 2.45	*< 0.001*
Microwave Ablation	-0.80	-2.44, 0.83	0.336

f) **Impact of transarterial chemoembolization on HCC-AD's performance.**

	**Coefficient**	**95% CI**	**p Value**
HCC-AD	1.78	0.89, 2.67	*< 0.001*
Transarterial Chemoembolization	-1.29	-2.73, 0.15	0.079

g) **Impact of chemotherapy on HCC-AD's performance.**

	**Coefficient**	**95% CI**	**p Value**
HCC-AD	1.69	0.84, 2.54	*< 0.001*
Chemotherapy	-0.48	-2.21, 1.25	0.589

7. Impact of aetiology on predictive capacity of HCC-AD score

Table S4: Prognostic capacity of HCC-AD score for 90-day mortality in patients when adjusted for aetiology of liver disease

	**Coefficient**	**95% CI**	**p Value**
HCC-AD	2.27	1.17, 3.36	*<0.001*
Alcohol-related liver disease	-2.24	-3.93, -0.55	*0.009*

	**Coefficient**	**95% CI**	**p Value**
HCC-AD	1.72	0.85, 2.58	*<0.001*
Hepatitis B virus	0.59	-1.46, 2.64	0.57

	**Coefficient**	**95% CI**	**p Value**
HCC-AD	1.66	0.83, 2.50	*<0.001*
Hepatitis C virus	0.01	-1.15, 1.17	0.99

	**Coefficient**	**95% CI**	**p Value**
HCC-AD	1.73	0.86, 2.59	*<0.001*
Metabolic dysfunction associated liver disease	1.69	-0.92, 4.30	0.20

8. Subgroup analysis of HCC-AD score for patient depending on the precipitant of decompensation.

Table S5: Prognostic capacity of HCC-AD score for 90-day mortality in patients with decompensation secondary to HCC progression

	**Coefficient**	**p-Value**
Decompensation related to HCC progression	2.80 (0.92, 4.68)	*0.003*
Other precipitant for decompensation	1.62 (1.12, 2.13)	*< 0.001*

9. **90-day mortality as predicted by the 7-day change in bilirubin.**

Figure S2: 90-day mortality as predicted by 7-day change in bilirubin. PPV = positive predictive value. NPV = negative predictive value

a)

Change in Bilirubin	Sensitivity	Specificity	PPV	NPV
≥ -10	80.52 (74.20, 86.84)	22.97 (16.26, 29.68)	52.10 (44.13, 60.07)	53.12 (45.17, 61.08)
≥ -5	75.32 (68.45, 82.20)	39.19 (31.40, 46.98)	56.31 (48.40, 64.22)	60.42 (52.62, 68.22)
≥ 0	67.53 (60.06, 75.00)	67.57 (60.10, 75.03)	68.42 (61.01, 75.84)	66.67 (59.15, 74.19)
≥ 5	61.04 (53.26, 68.82)	83.78 (77.90, 89.66)	79.66 (73.90, 86.08)	67.39 (59.91, 74.87)
≥ 10	50.65 (42.68, 58.62)	91.89 (87.54, 96.25)	86.67 (81.24, 92.09)	64.15 (56.50, 71.80)

b)

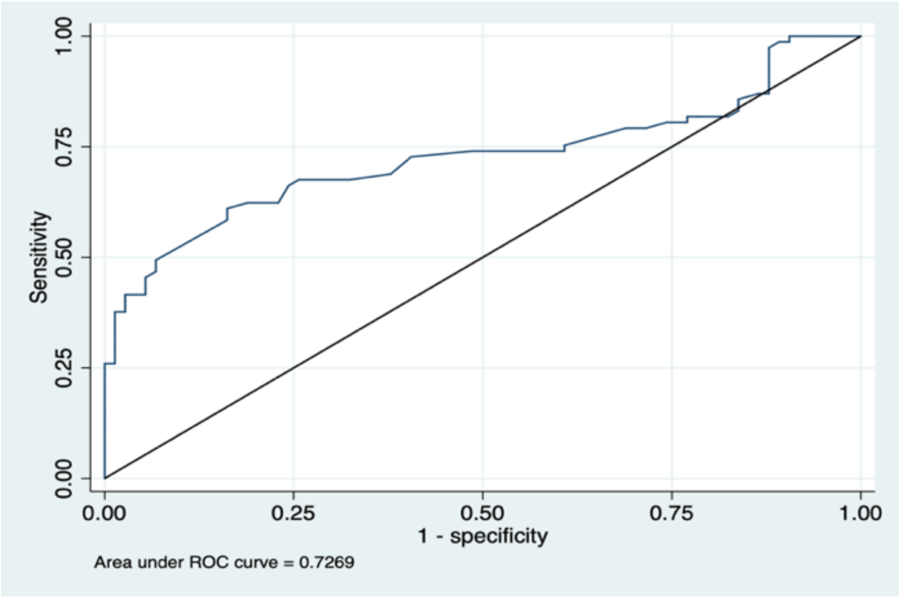

10. **90-day mortality as predicted by 7-day change in MELD 3.0 score.**

Figure S3: 90-day mortality as predicted by 7-day change in MELD 3.0 score. PPV = positive predictive value. NPV = negative predictive value

a)

Change in MELD 3.0	Sensitivity	Specificity	PPV	NPV
≥ -2	73.53 (65.76, 81.29)	17.86 (11.12, 24.60)	52.08 (43.29, 60.88)	35.71 (27.28, 44.15)
≥ -1	61.76 (53.21, 70.32)	23.21 (15.78, 30.65)	49.41 (40.61, 58.21)	33.33 (25.04, 41.63)
≥ 0	50.00 (41.20, 58.80)	35.71 (27.28, 44.15)	48.57 (39.77, 57.37)	37.04 (28.54, 45.54)
≥ 1	38.24 (29.68, 46.79)	53.57 (44.79, 62.35)	50.00 (41.20, 58.80)	41.67 (32.99, 50.34)
≥ 2	30.88 (22.75, 39.01)	64.29 (55.85, 72.72)	51.22 (42.42, 60.02)	43.37 (34.65, 52.10)

b)

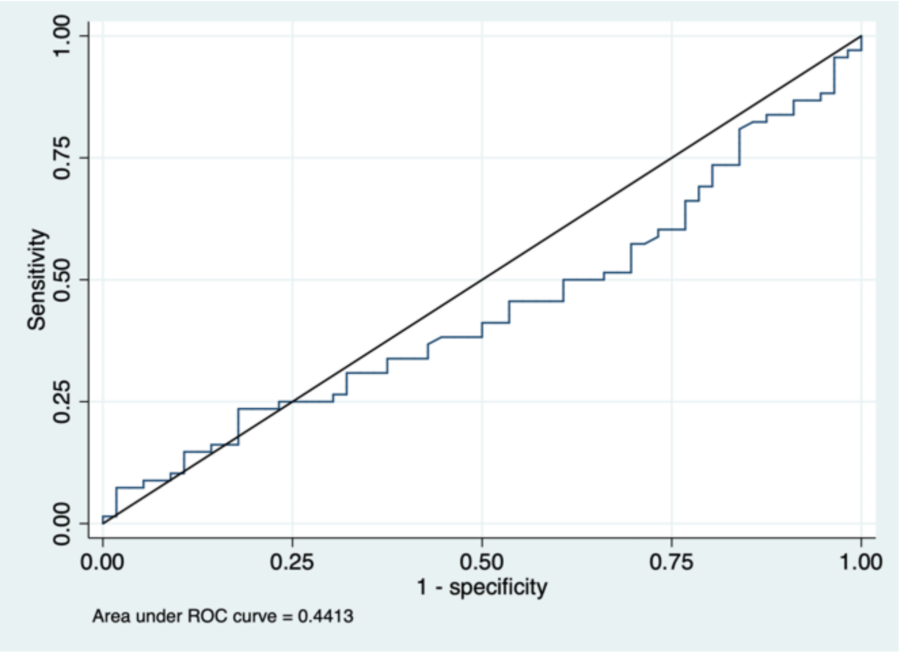

11. **90-day mortality as predicted by 7-day change in ALBI Score.**

Figure S4: 90-day mortality as predicted by 7-day change in ALBI Score. PPV = positive predictive value. NPV = negative predictive value

a)

Change in ALBI	Sensitivity	Specificity	PPV	NPV
≥ -1	96.10 (93.20, 99.19)	5.41 (1.80, 9.01)	51.39 (43.42, 59.36)	57.14 (49.25, 65.04)
≥ -0.5	75.32 (68.45, 82.20)	14.86 (9.19, 20.54)	47.93 (39.97, 55.90)	36.67 (28.98, 44.35)
≥ 0	35.06 (27.45, 42.68)	62.16 (54.43, 69.90)	49.09 (41.12, 57.06)	47.92 (39.95, 55.88)
≥ 0.2	20.78 (14.31, 27.25)	81.08 (74.83, 87.33)	53.33 (45.38, 61.29)	49.59 (41.61, 57.56)
≥ 0.5	7.79 (3.52, 12.07)	95.95 (92.80, 99.09)	66.67 (59.15, 74.19)	50.00 (42.03, 57.97)

b)

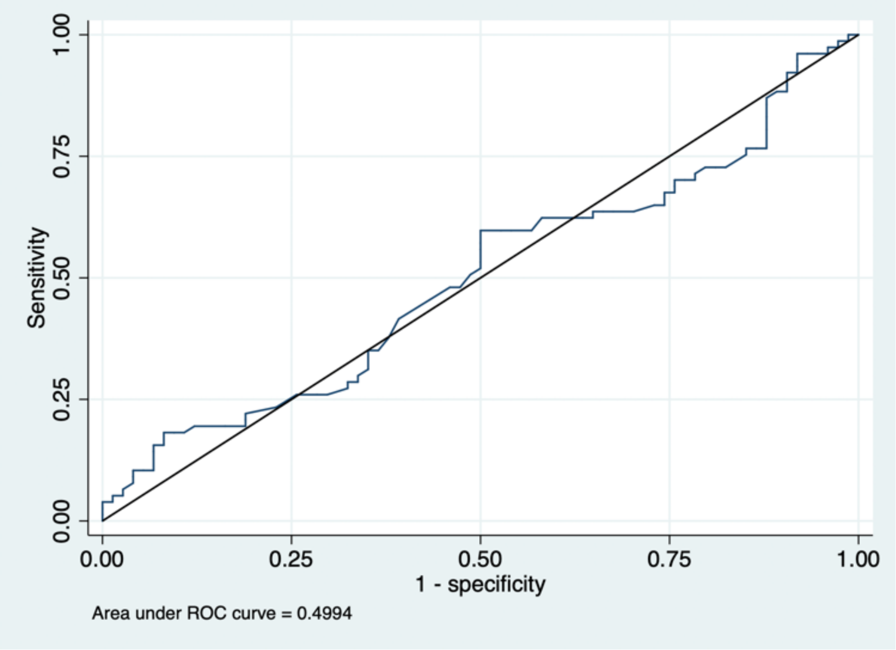

12. **90-day mortality as predicted by 7-day change in ALBI Score.**

Figure S5: 90-day mortality as predicted by 7-day change in Child Pugh Score. PPV = positive predictive value. NPV = negative predictive value

a)

Change in Child Pugh Score	Sensitivity	Specificity	PPV	NPV
≥ 0	82.61 (75.96, 89.25)	14.29 (8.15, 20.42)	54.29 (45.55, 63.02)	40.00 (31.41, 48.59)
≥ 1	40.58 (31.97, 49.19)	58.93 (50.30, 67.55)	54.90 (46.18, 63.62)	44.59 (35.88, 53.31)
≥ 2	5.80 (1.70, 9.89)	85.71 (79.58, 91.85)	33.33 (25.07, 41.60)	42.48 (33.81, 51.14)

b)

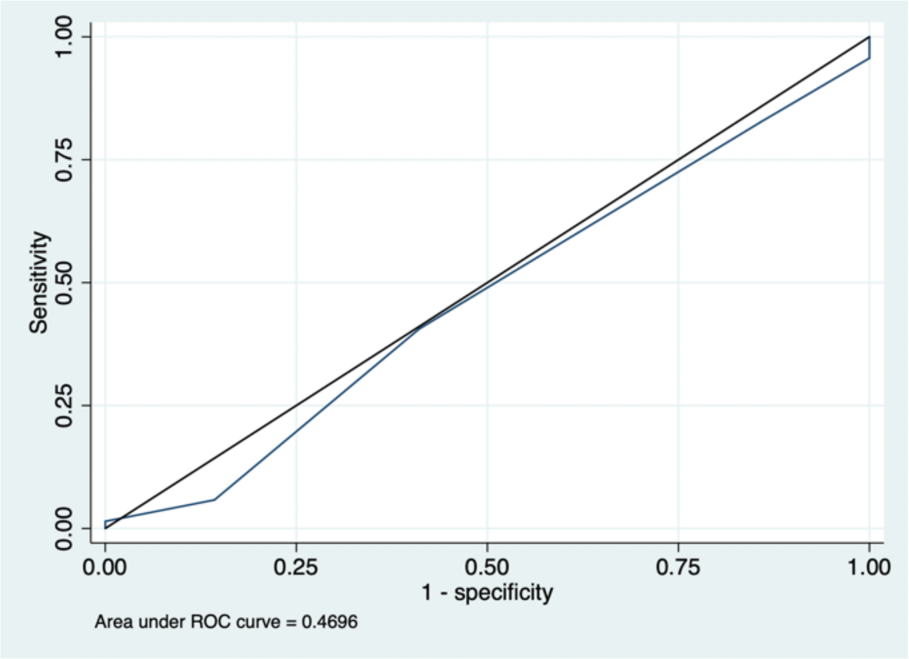

13. **Observed vs predicted mortality based on HCC-AD group in validation cohort**

Figure S6: Observed and predicted mortality in validation group based on HCC-AD group

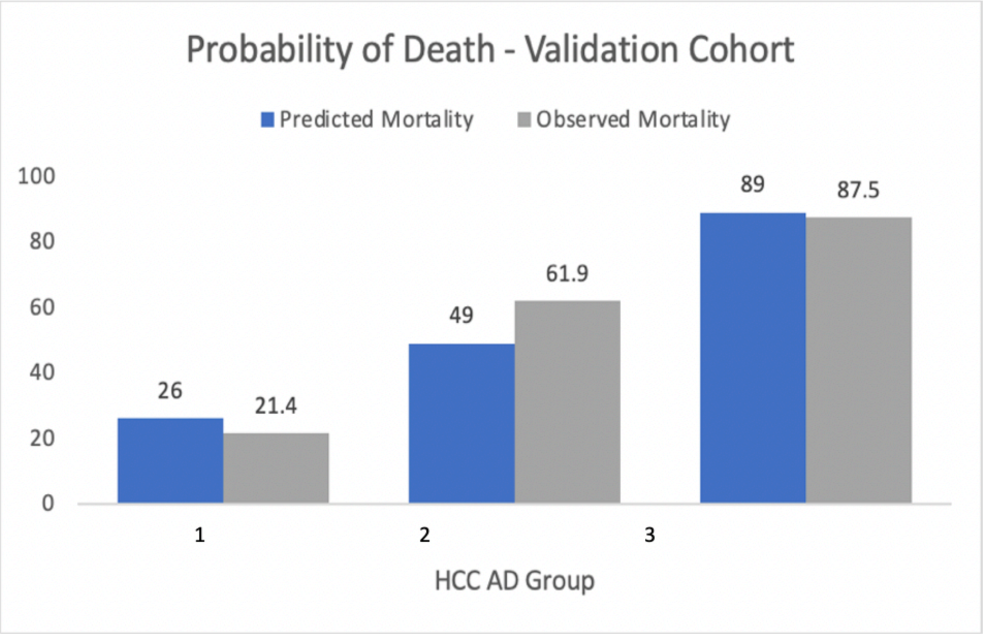

14. **AUROC of various scores**

Figure S7: Area under the receiver operating characteristic curve (AUROC) of the HCC-AD, the MELD 3.0 score, Child Pugh score and the ALBI score

a)

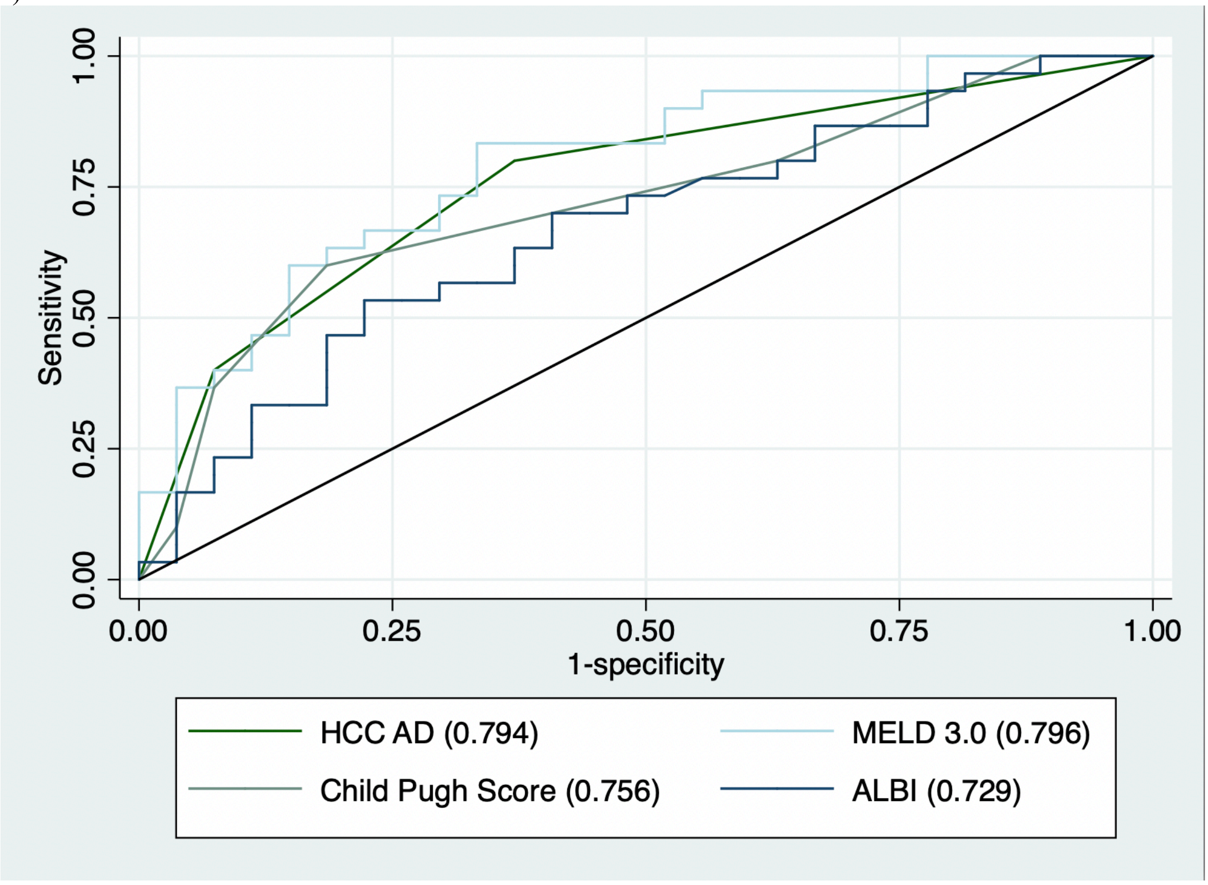

b)

	AUC	Standard Error	95% CI
HCC AD	0.794	0.053	0.690, 0.897
MELD 3.0	0.796	0.042	0.712, 0.879
Child Pugh	0.756	0.042	0.675, 0.834
ALBI	0.729	0.045	0.641, 0.817

15. **Observed vs predicted survival based on HCC-AD**

Figure S8: Observed vs predicted survival of the three risk groups as stratified by HCC-AD

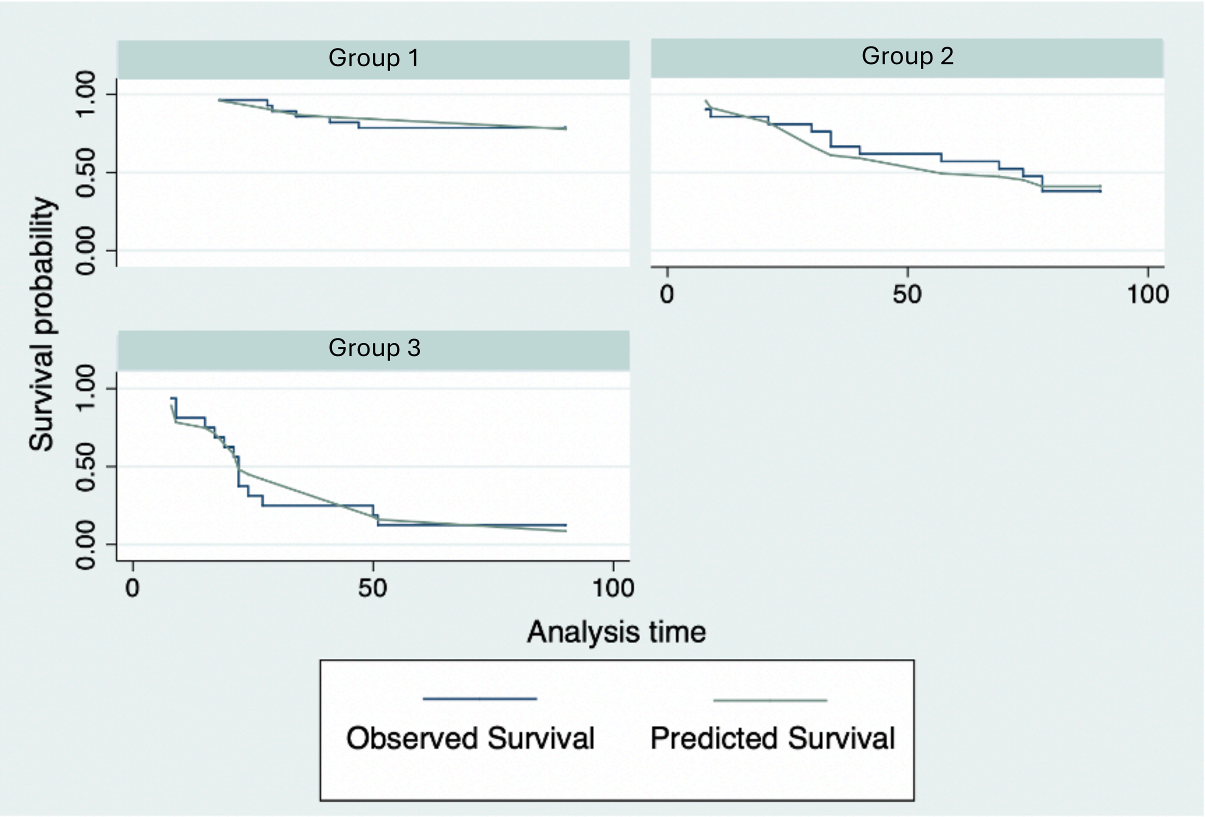

Supplement: Supplementary Material [file mmc1.pdf]
